# Double deficiency of Trex2 and DNase1L2 nucleases leads to accumulation of DNA in lingual cornifying keratinocytes without activating inflammatory responses

**DOI:** 10.1038/s41598-017-12308-4

**Published:** 2017-09-19

**Authors:** Joan Manils, Heinz Fischer, Joan Climent, Eduard Casas, Celia García-Martínez, Jordi Bas, Supawadee Sukseree, Tanya Vavouri, Francisco Ciruela, Josep Maria de Anta, Erwin Tschachler, Leopold Eckhart, Concepció Soler

**Affiliations:** 1 Departament de Patologia i Terapèutica Experimental, Facultat de Medicina i Ciències de la Salut, IDIBELL, Universitat de Barcelona, L’Hospitalet de Llobregat, Barcelona, Spain; 2 Research Division of Biology and Pathobiology of the Skin, Department of Dermatology, Medical University of Vienna, Vienna, Austria; 3 Departament d’Immunologia, Hospital Universitari de Bellvitge, L’Hospitalet de Llobregat, Barcelona, Spain; 4 Program of Predictive and Personalized Medicine of Cancer (PMPPC) - Institute Germans Trias i Pujol (IGTP), Badalona, Barcelona, Spain; 5 Josep Carreras Leukaemia Research Institute (IJC), ICO-Hospital Germans Trias i Pujol, Badalona, Barcelona, Spain; 6 Present Address: The Francis Crick Institute-Mill Hill Laboratory, London, NW7 1AA United Kingdom; 7 Present Address: Unit of Pathology of Laboratory Animals, University of Veterinary Medicine, Vienna, Austria

## Abstract

The cornification of keratinocytes on the surface of skin and oral epithelia is associated with the degradation of nuclear DNA. The endonuclease DNase1L2 and the exonuclease Trex2 are expressed specifically in cornifying keratinocytes. Deletion of DNase1L2 causes retention of nuclear DNA in the tongue epithelium but not in the skin. Here we report that lack of Trex2 results in the accumulation of DNA fragments in the cytoplasm of cornifying lingual keratinocytes and co-deletion of DNase1L2 and Trex2 causes massive accumulation of DNA fragments throughout the cornified layers of the tongue epithelium. By contrast, cornification-associated DNA breakdown was not compromised in the epidermis. Aberrant retention of DNA in the tongue epithelium was associated neither with enhanced expression of DNA-driven response genes, such as *Ifnb*, *Irf7* and *Cxcl10*, nor with inflammation. Of note, the expression of *Tlr9*, *Aim2* and *Tmem173*, key DNA sensor genes, was markedly lower in keratinocytes and keratinocyte-built tissues than in macrophages and immune tissues, and DNA-driven response genes were not induced by introduction of DNA in keratinocytes. Altogether, our results indicate that DNase1L2 and Trex2 cooperate in the breakdown and degradation of DNA during cornification of lingual keratinocytes and aberrant DNA retention is tolerated in the oral epithelium.

## Introduction

Cornification is a mechanistically unique mode of programmed cell death that is restricted to keratinocytes undergoing terminal differentiation. Keratinocytes form multilayered squamous epithelia on the body surface and its invaginations such as the oral cavity. Within these epithelia, cells proliferate exclusively in the basal layer and undergo progressive differentiation in the suprabasal layers. Keratinocyte differentiation is associated with tightly regulated sequential changes in gene expression, modifications of proteins, and synthesis of particular lipids that build a physical barrier between the organism and the environment. Abnormalities in many cornification components result in enhanced transepidermal water loss, compromise the defence against infections, and underlie the development of several skin diseases. Distinct cornification programs give rise to the epidermis of the skin, oral and urogenital epithelia, and specialized skin appendages, such as hair, nails, and filiform papillae of the tongue, thereby conferring singular properties to each epithelium^1–3^.

Nuclear destruction is one of the most profound changes of the cell during cornification. Delay or absence of this breakdown process results in parakeratosis, which is found in a myriad of skin diseases, such as psoriasis and atopic dermatitis^4^, and also appears during wound healing^5^. While the appearance of parakeratosis in diverse skin disorders suggests that the degradation of the nucleus can be disturbed by multiple molecular triggers, the molecular regulation of cornification-associated breakdown of nuclear components and the etiological roles of these processes have remained poorly characterized. Phosphorylation of lamin A/C by Akt1^6^, the nuclear transfer of profilaggrin fragments, and proteolytic activities of caspase-14^7–9^ have been implicated in the control of keratinocyte enucleation.

The fragmentation and degradation of nuclear DNA are hallmarks of programmed cell death. Specific enzymes (DNases) degrade DNA in different physiological settings of cell death and, to a limited extent, during DNA repair^10–12^. DNases are also involved in the process of DNA degradation during cornification of keratinocytes. The keratinocyte-specific endonuclease DNase1L2 is required for nuclear DNA degradation in human keratinocytes differentiating in skin models *in vitro* ^13^. Knockout of DNase1L2 in the mouse revealed a crucial role of DNase1L2 in DNA degradation in terminally differentiated keratinocytes of hair, nails, tail scales, filiform papillae of the tongue, and in the esophagus^14^. Deletion of the lysosomal endonuclease DNase2 impaired DNA degradation during holocrine secretion of sebum but did not abrogate cornification-associated DNA breakdown in the interfollicular epidermis^15,16^. Only the deletion of both DNase1L2 and DNase2 blocked the removal of nuclear DNA during stratum corneum formation in the epidermis, suggesting that DNase1L2 and DNase2 cooperate in cornifying epidermal keratinocytes^17^. Trex2 is a 3′-exonuclease which is predominantly expressed in keratinocytes and contributes to the epidermal response to UVB-induced DNA damage^18^. Deficiency of Trex2 increased the susceptibility to skin carcinogenesis in mice^18,19^. Deletion of Trex2 also enhanced parakeratosis in the imiquimod-induced model of psoriasis in mice^20^. As the loss of Trex2 alone did not induce parakeratosis under tissue homeostasis but predisposed to parakeratosis under exposure to pro-inflammatory stress^20^, a cell-autonomous role of Trex2 in keratinocytes under non-inflammatory conditions remained elusive.

The blockade of DNA degradation by inactivation of the lysosomal endonuclease DNase2^11,21^ or the loss of endonucleases responsible for extracellular DNA degradation, i.e. DNase1^22,23^ and DNase1L3^24,25^, results in the aberrant accumulation of self-DNA originating from dying cells, leading to over-activation of immune responses and subsequent onset of autoimmune diseases. Similarly, the loss of the exonuclease Trex1, which degrades DNA in the cytosol of many cell types, results in DNA accumulation leading to the induction of type I interferon (IFN) responses and autoimmunity^26–30^. The suppression of the nuclear DNA breakdown in cornifying keratinocytes does not induce skin inflammation^14,16^. Therefore, the lack of a tissue response to aberrantly retained DNA appears to be caused either by a suppressive mechanism that is associated with differentiated keratinocytes, such as Trex2-mediated degradation of DNA leaking into the cytosol, or by a putative lack of DNA-sensing and response factors in differentiated keratinocytes.

In the present study we investigated the effects of concomitant suppression of DNase1L2 and Trex2 on DNA degradation during cornification and on DNA-dependent inflammation. Taking into account that Trex2 processes DNA from 3′-OH ends^31^ that can be generated by DNase1-type nucleases, such as DNase1L2^13^, we hypothesized that Trex2 and DNase1L2 might cooperate in DNA degradation. We demonstrate that there is an interplay between these two nucleases in lingual keratinocytes and accumulation of cytosolic DNA fragments in the absence of Trex2 and DNase1L2. Furthermore, our results show that homeostatic keratinocytes express only low levels of various DNA response genes and display tolerance to aberrantly retained endogenous DNA.

## Results

### The Trex2 exonuclease and the DNase1L2 endonuclease are expressed in differentiated keratinocytes

**Figure 1:**
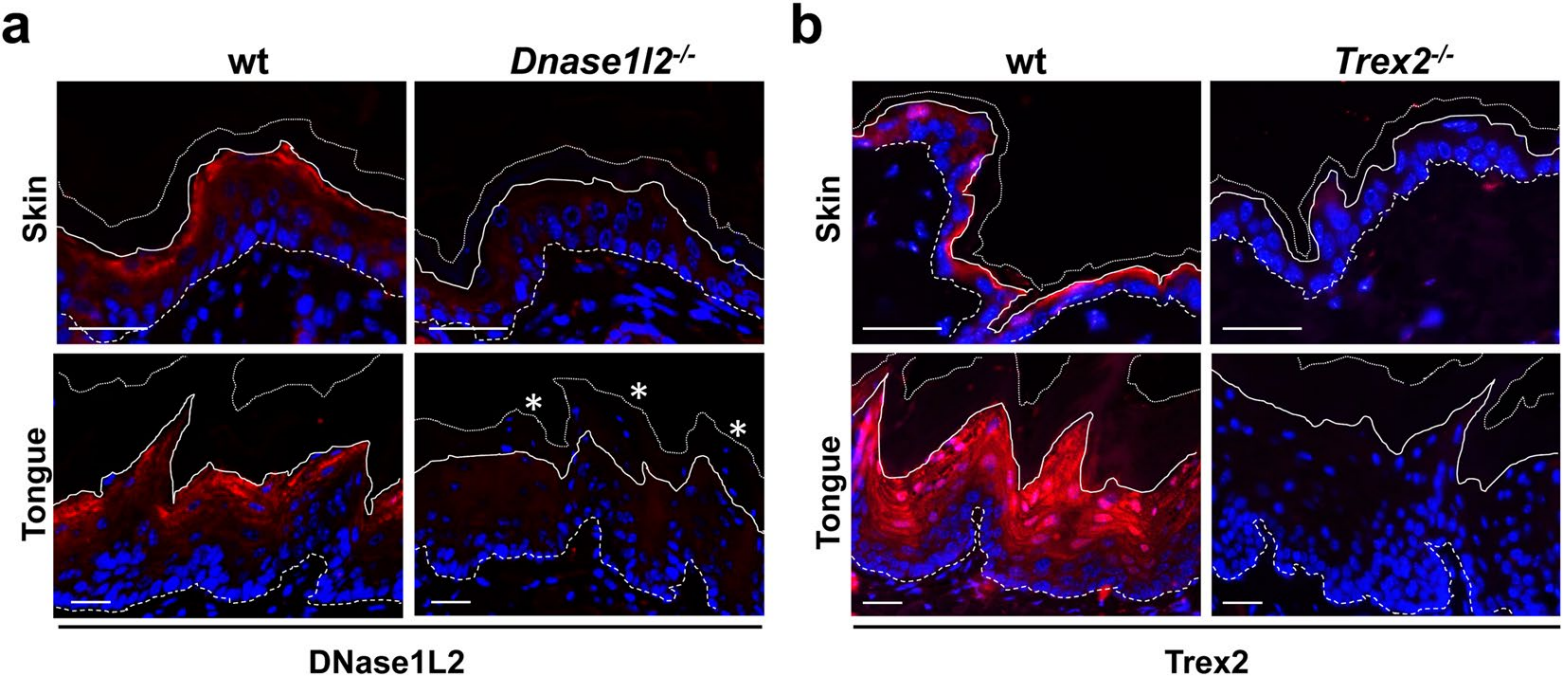
The nucleases Trex2 and DNase1L2 are mostly expressed in the keratinocyte upper layers of the skin and tongue epithelia. (**a**) Immunofluorescence images showing DNase1L2 (red) and DNA (blue) staining in the back skin and tongue sections from wild-type (wt) and *Dnase1l2* knockout (*Dnase1l2* ^−/−^) mice. (**b**) Immunofluorescence images illustrating Trex2 (red) and DNA (blue) staining in back skin and tongue sections from wt and *Trex2* knockout (*Trex2* ^−/−^) mice. Scale bars = 25 μm. Dashed lines, epidermal-dermal border; continuous lines, bottom of the stratum corneum; dotted lines, upper border of the stratum corneum.

Among the multiple genes displaying DNA nuclease activity, the Trex2 exonuclease and the endonuclease DNase1L2 are specifically expressed in keratinocytes^13,14,18,19^. As shown in Fig. 1, immunofluorescence analysis revealed very similar patterns of expression of Trex2 and DNase1L2 proteins in differentiated keratinocytes of stratified squamous epithelia of both skin and tongue. The absence of staining in tissue samples from *Trex2* and *Dnase1l2* knockout mice confirmed the specificity of the immunolabelling reactions. Notably, the expression of both Trex2 and DNase1L2 proteins was increased in the upper keratinocyte layers, where endogenous DNA is degraded in the course of cornification. The distribution of DNase1L2 and Trex2 proteins in the epidermal layers correlated with the distribution of DNase1L2 and Trex2 mRNAs, that we obtained by analysis of the recently available transcriptomes of murine epidermal layers^32^. The mRNA levels of *Trex2* as well as *Dnase1l2* were highly upregulated specifically in the granular layer, whereas those of *Trex1* and *Dffb*/CAD were downregulated, compared to their expression in the basal layer. Even though to less extend, the mRNA levels of nucleases *Dnase1l3* and *Dnase2a* were also increased in the granular layer (Supplementary Figure S1). Consistently with previous studies^33,34^, epidermal differentiation was associated with increased expression of keratinocyte-specific *Casp14* and decreased expression of the canonical apoptotic protease caspase-3 at the mRNA level (Supplementary Figure S1). Analysis of nuclease gene expression in available transcriptomes of wounded epithelia^35^ revealed that injury of the skin led to a decrease of the mRNA level of *Dnase1l2* and to an increase of the expression levels of *Trex2* whereas no changes were observed upon injury of the tongue (Supplementary Figure S2). These data suggest that the expression of DNase1L2 and Trex2 is regulated by different mechanisms in response to skin wounding but apparently in a similar manner in homeostatic epithelia of the skin and the tongue.

### Trex2 and DNase1l2 collaborate in the DNA degradation of lingual keratinocytes undergoing terminal differentiation

**Figure 2:**
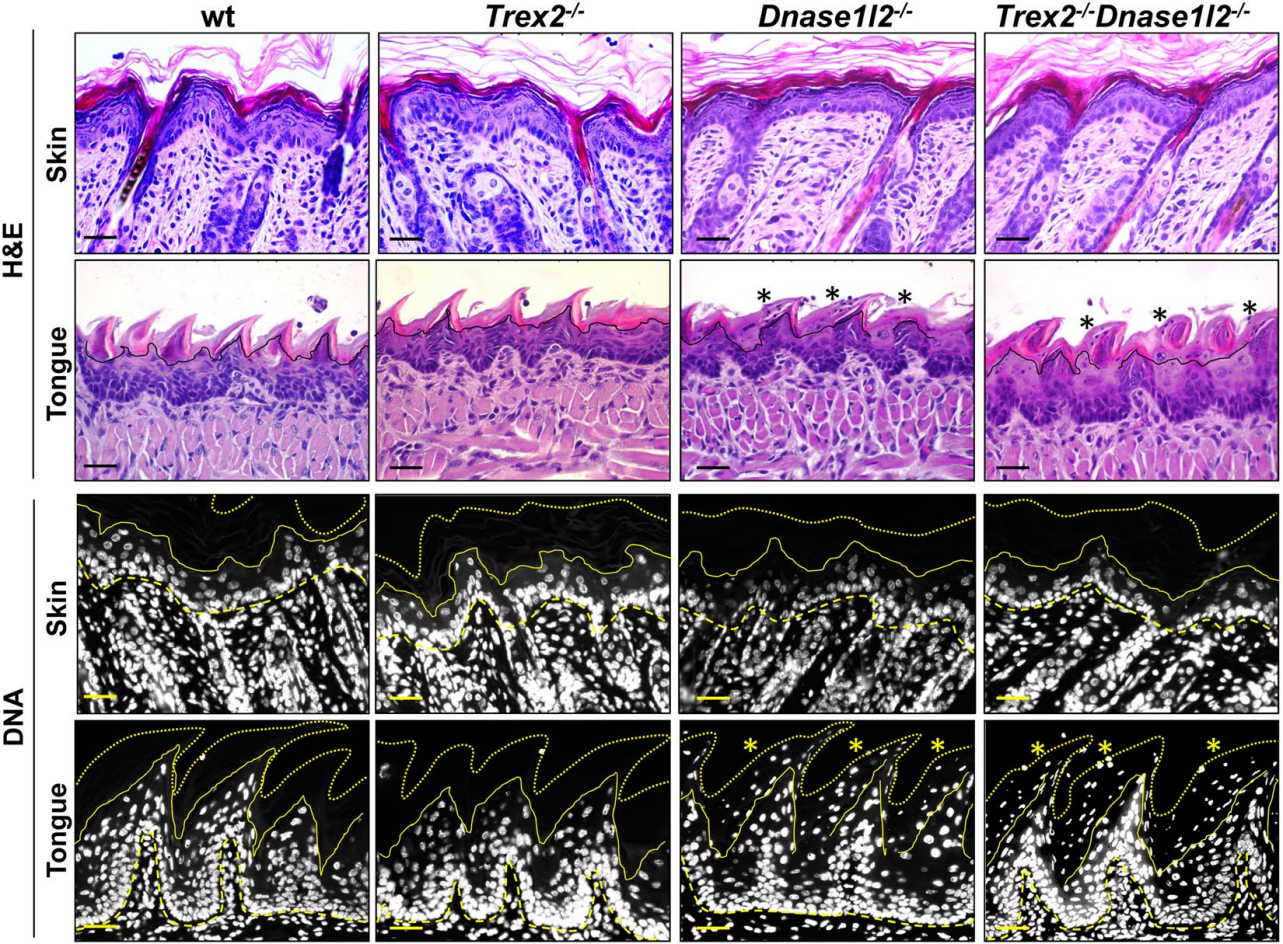
Double *Trex2* and *Dnase1l2* deficiency does not trigger parakeratosis in the skin. H&E (lanes 1 and 2) staining and Hoechst DNA labelling (lanes 3 and 4) in back skin and tongue sections from wt, *Trex2* ^−/−^, *Dnase1l2* ^−/−^ and *Trex2* ^−/−^ *Dnase1l2* ^−/−^ mice. Back skin samples were from 6 days-old mice which are consistently in the anagen phase of the hair cycle. Likewise, adult back skin was free from parakeratosis in all samples investigated. Tongue samples were from adult 7–9 weeks-old mice. The images are representative for at least five mice from each genotype. Scale bars = 25 μm. Dashed lines, epidermal-dermal border; continuous lines, bottom of the stratum corneum; dotted lines, upper border of the stratum corneum.

Fluorescent dye labelling of DNA (Fig. 1a and Fig. 2) and H&E staining (Fig. 2) showed that *Dnase1l2* deficiency in mice led to retention of nuclei in the stratum corneum of the tongue (asterisks) but not of the skin, confirming our previous report^14^ that DNase1L2 was indispensable in the tongue epithelium whereas nuclear DNA degradation could proceed to completion even in the absence of DNase1L2 in cornifying epidermal keratinocytes. By contrast, DNA-specific fluorescent dye labelling showed that the loss of *Trex2* did not result in aberrant DNA abundance in the stratum corneum of either skin^18,19^ or tongue under normal situations (Figs 1b and 2), suggesting that Trex2 action would be dispensable for most of nuclear DNA degradation. Nevertheless, this might partially be due to compensatory mechanisms.

To determine whether the keratinocyte-specific Trex2 and DNase1L2 nuclease activities cooperate and/or overlap throughout the DNA degradation process leading to keratinocyte enucleation, we generated Trex2 Dnase1l2 (*Trex2* ^−/−^ *Dnase1l2* ^−/−^) double knockout mice by crossing *Trex2* (*Trex2* ^−/−^)^18^ and *Dnase1l2* (*Dnase1l2* ^−/−^)^14^ knockout mice. The *Trex2* ^−/−^ *Dnase1l2* ^−/−^ mice were viable, born at the expected Mendelian frequency and indistinguishable by weight and growth from their double-heterozygous and single gene knockout littermates. These mice seemed healthy and no apparent skin abnormalities or an increase in skin diseases such as skin hyperpigmentation, alopecia, dryness and dermatitis, or spontaneous skin tumours were observed up to one year of age. Therefore, simultaneous loss of *Trex2* and *Dnase1l2* genes does not compromise survival into adulthood and does not generate an evident skin-related disease phenotype. Likewise the tongue was macroscopically inconspicuous in *Trex2* ^−/−^ *Dnase1l2* ^−/−^ mice. The numbers of living keratinocyte layers in the skin and lingual epithelia were not altered upon deletion of either *Dnase1l2*, *Trex2* or both, suggesting that the inactivation of these DNases did not affect the cell turnover rates in epithelia.

To analyse the effects of the combined *Trex2* and *Dnase1l2* deficiencies on nuclear destruction during keratinocyte cornification, we first performed H&E staining and fluorescent labelling of DNA in skin and tongue samples from wild-type, *Trex2* ^−/−^, *Dnase1l2* ^−/−^ and *Trex2* ^−/−^ *Dnase1l2* ^−/−^ mice. We did not detect abnormal retention of nuclei in the stratum corneum of interfollicular epidermis of back, ear and snout skin from *Trex2* ^−/−^ *Dnase1l2* ^−/−^, *Dnase1l2* ^−/−^ and *Trex2* ^−/−^ mice by H&E staining and fluorescence labelling with a DNA-specific dye (Fig. 2, Supplemental Figure S3), indicating that neither single nor simultaneous loss of Trex2 and DNase1L2 prevented the removal of nuclei in cornifying epidermal keratinocytes. The lingual filiform papillae of *Dnase1l2* ^−/−^ and *Trex2* ^−/−^ *Dnase1l2* ^−/−^ mice showed an aberrant accumulation of nuclear remnants in the cornified cell layers which was not observed in the corresponding samples of *Trex2* ^−/−^ and wild-type mice (Fig. 2, asterisks), suggesting that loss of DNase1L2^14^ but not loss of Trex2 leads to parakeratosis in lingual epithelia.

**Figure 3:**
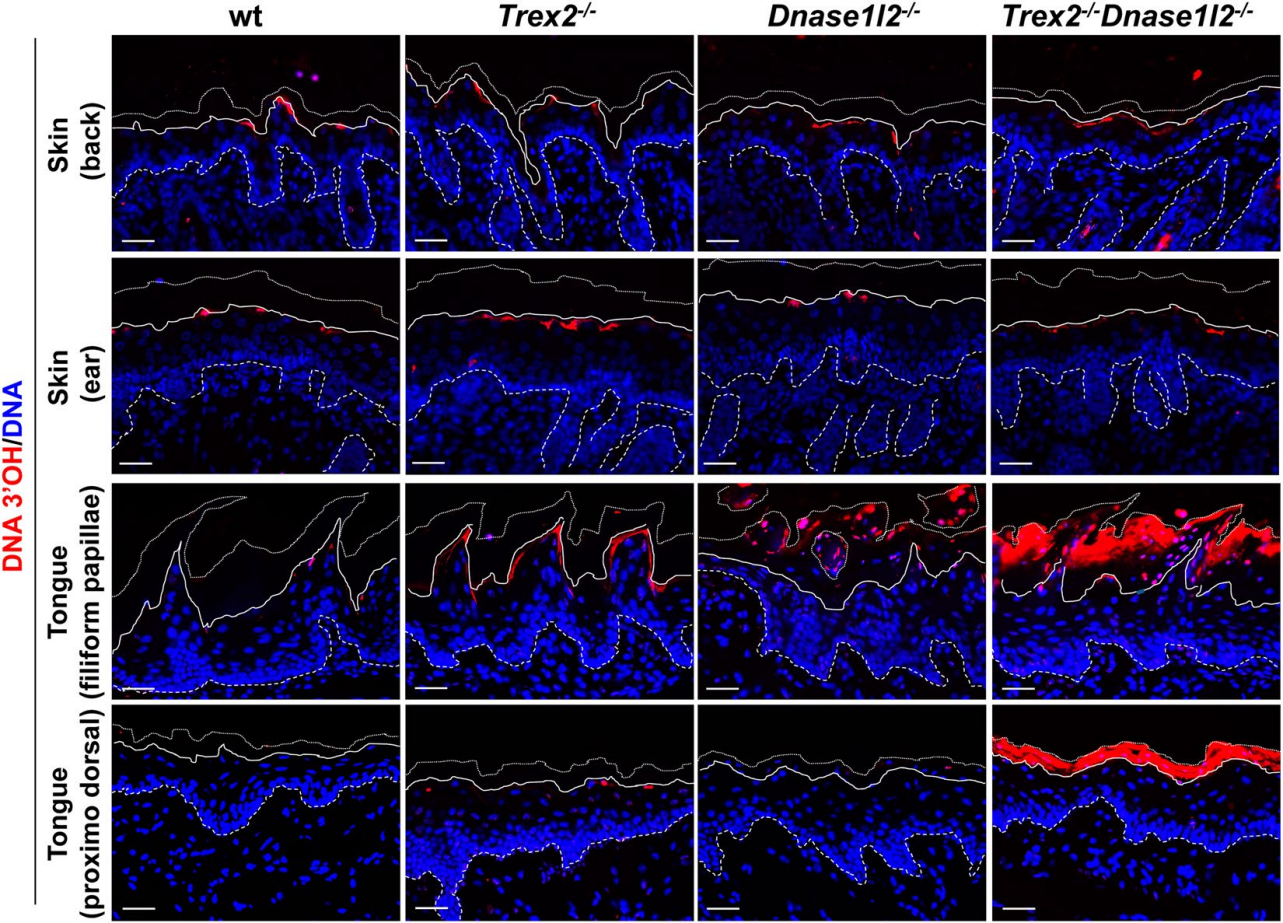
Double *Trex2* and *Dnase1l2* deficiency leads to an increase of fragmented DNA in the upper keratinocyte layers of the tongue, but not of the epidermis. TUNEL labelling of free 3′-OH DNA ends (red) and nuclei (blue) counterstained with Hoechst of back and ear skin, and the filiform papillae and back side tongue sections from wt, *Trex2* ^−/−^, *Dnase1l2* ^−/−^ and *Trex2* ^−/−^ *Dnase1l2* ^−/−^ mice. Back and ear skin samples were from 6 days-old mice. Tongue samples were from 7–9 weeks-old mice. The images are representative for at least five mice from each genotype. Scale bars = 25 μm. Dashed lines, epidermal-dermal border; continuous lines, bottom of the stratum corneum; dotted lines, upper border of the stratum corneum.

Next, we analysed the skin and tongue epithelia using the highly sensitive TUNEL assay for the presence of DNA fragments with free 3′-OH ends. In accordance with previous studies^14,19,20^, TUNEL-positive nuclei were observed sporadically in the epidermis of wild-type, *Trex2* ^−/−^, *Dnase1l2* ^−/−^ and *Trex2* ^−/−^ *Dnase1l2* ^−/−^ mice. These nuclei were confined to epidermal keratinocytes in the transition stage from stratum granulosum to stratum corneum, indicating that DNA degradation during epidermal cornification involves TUNEL-positive intermediates both in the presence and absence of DNase1L2 and Trex2. In contrast to the uniform TUNEL labelling in the epidermis of all mouse strains investigated, there were profound differences in the TUNEL labelling in the cornified cell layers of the lingual stratum corneum of wild-type, *Trex2* ^−/−^, *Dnase1l2* ^−/−^, and *Trex2* ^−/−^ *Dnase1l2* ^−/−^ mice. TUNEL-positive nuclei were present in cornifying lingual keratinocytes of wild-type mice in a pattern similar to that observed in the epidermis (Fig. 3). Deletion of *Trex2* resulted in the presence of TUNEL-positive DNA fragments not only in nucleus but also in the cytoplasm of lingual keratinocytes undergoing the transition from the living to cornified layer of the filiform papillae. In DNase1L2-deficient mice nuclear remnants within the parakeratotic filiform papillae were TUNEL-positive. The combined loss of *Trex2* and *Dnase1l2* was associated with the strong accumulation of fragmented DNA in the nucleus and in the cytosol of cornifying cells and in the entire stratum corneum (Fig. 3, panels in the third row). Lingual epithelium without filiform papillae displayed only sporadic TUNEL signals in wild-type, *Trex2* ^−/−^, and *Dnase1l2* ^−/−^ mice but strong accumulation of TUNEL-positive DNA fragments, similar to that observed in filiform papillae, in *Trex2* ^−/−^ *Dnase1l2* ^−/−^ mice (Fig. 3, panels in the fourth row). These results indicated that (1) TUNEL-positive DNA fragments could be formed in a DNase1L2 and Trex2-independent manner during lingual keratinocyte cornification, and (2) Trex2 critically contributed to the degradation of nuclear DNA leaking into the cytosol of cornifying keratinocytes. Thus, cytosolic DNA fragments were confined to the transition cells of filiform papillae when only Trex2 was missing, suggesting that during normal cornification these fragments were reduced by Trex2 to levels below the detection limit of our assay. By contrast, when both Trex2 and DNase1L2 were absent, DNA fragments were either retained or further released from nuclear remnants when cornified cells moved towards the surface of the epithelium.

### Absence of immune response to aberrantly accumulated DNA in lingual differentiated keratinocytes

**Figure 4:**
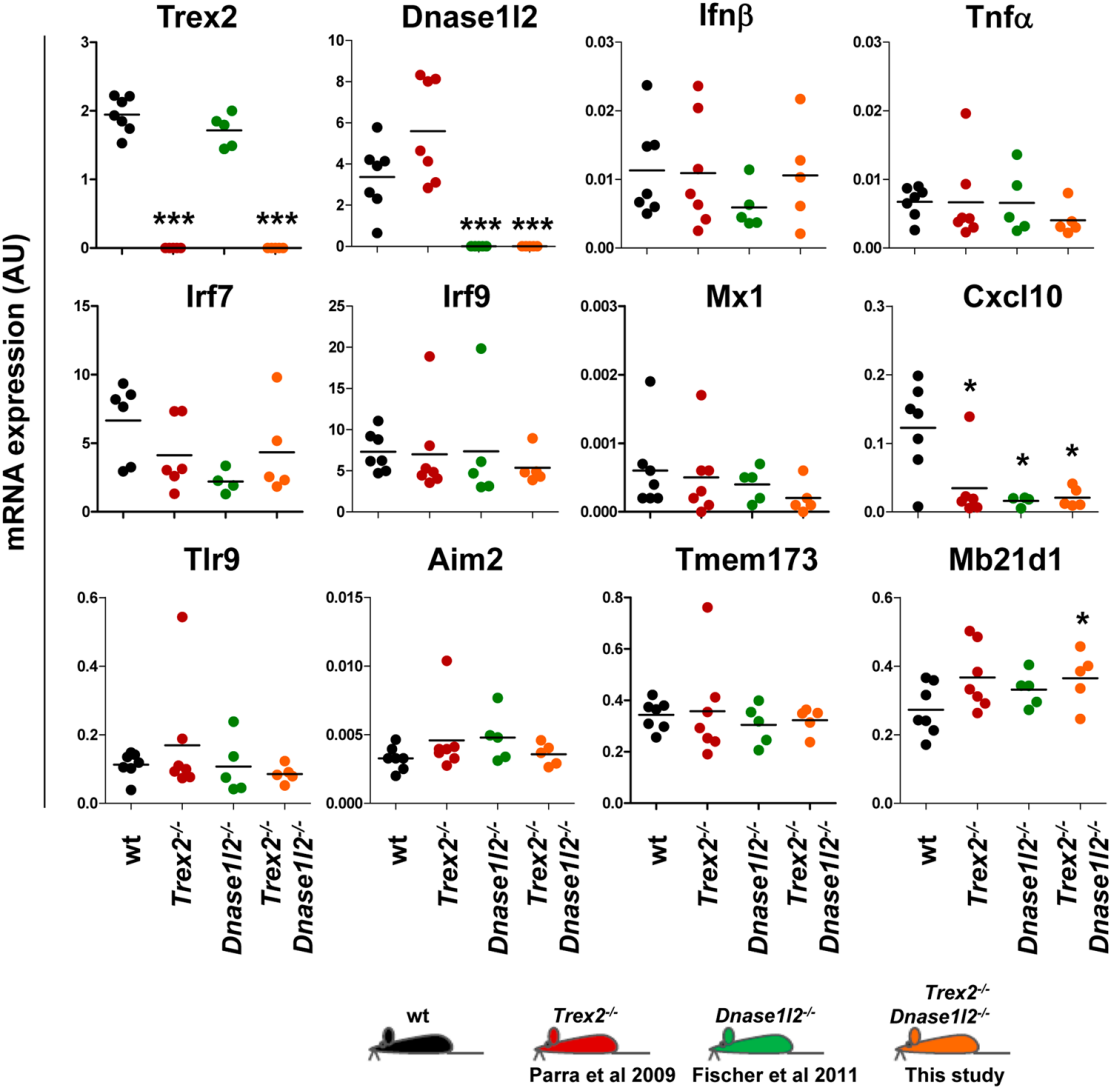
DNA-driven gene expression signature is not induced in the tongue of the *Trex2* ^−/−^ *Dnase1l2* ^−/−^ mice. Expression of the indicated genes in the tongue from wt, *Trex2* ^−/−^, *Dnase1l2* ^−/−^ and *Trex2* ^−/−^ *Dnase1l2* ^−/−^ mice, as determined by RT-qPCR. Each dot indicates a sample from an individual mouse, and horizontal lines represent the mean value. The results from one of two independent experiments with similar results are shown. Significant differences, by the Mann-Whitney test between genotypes: *P **<** 0.05; ***P **<** 0.001. AU, arbitrary units.

Intriguingly, in spite of the huge amount of cytosolic DNA aberrantly accumulated in the upper keratinocyte layers of the *Trex2* ^−/−^ *Dnase1l2* ^−/−^ tongue, no signs of inflammation and immune cell infiltration were histologically observed by H&E staining (Fig. 2), and no straight transcriptional signature of DNA-driven genes was found by RT-qPCR analysis (Fig. 4). In particular, although a significant, slight increase in the Mb21d1 (cGAS) mRNA expression levels was observed in *Trex2* ^−/−^ *Dnase1l2* ^−/−^ mice compared to wild-type mice, double deficiency of *Trex2* and *Dnase1l2* in the tongue did not induce major type I IFN and IFN-inducible genes, including *Ifnβ*, *Irf7*, *Irf9*, *Mx1*, *Tlr9*, and *Aim2*, or inflammatory genes, such as *Tnf*; mRNA levels of Cxcl10 were even lower in *Trex2* ^−/−^ *Dnase1l2* ^−/−^ mice than in wild-type mice. Furthermore, no significant changes in the mRNA levels of the keratinocyte differentiation genes *Flg*, *Lor*, *Ivl* and *Krt10* were observed by single or double loss of the Trex2 and DNase1L2 nucleases (Supplementary Figure S4). Therefore, disrupted DNA degradation during lingual cornification does not result in the deregulation of the expression of key genes related to either DNA-driven responses or keratinocyte differentiation. Also, by indirect immunofluorescence analysis of antinuclear antibodies (ANAs), antibodies binding nuclear mitotic apparatus (NUMA) and nucleolus were detected in one out of five *Trex2* ^−/−^ *Dnase1l2* ^−/−^ mice and one out of five *Dnase1l2* ^−/−^ mice, respectively (Supplementary Figure S5). Nevertheless, immuno-dot-blot analysis did not indicate increased serum levels of antibodies against chromatin antigens, such as nucleosomes and histones, in *Dnase1l2* ^−/−^ or *Trex2* ^−/−^ *Dnasel12* ^−/−^ mice compared to *Trex2* ^−/−^ and wild-type mice. Thus, single or double absence of keratinocyte-specific *Trex2* and *Dnase1l2* may trigger an immune response to nuclear-associated structures, but did not clearly result in DNA-driven immune response activation, even when defects in DNA degradation were evident.

**Figure 5:**
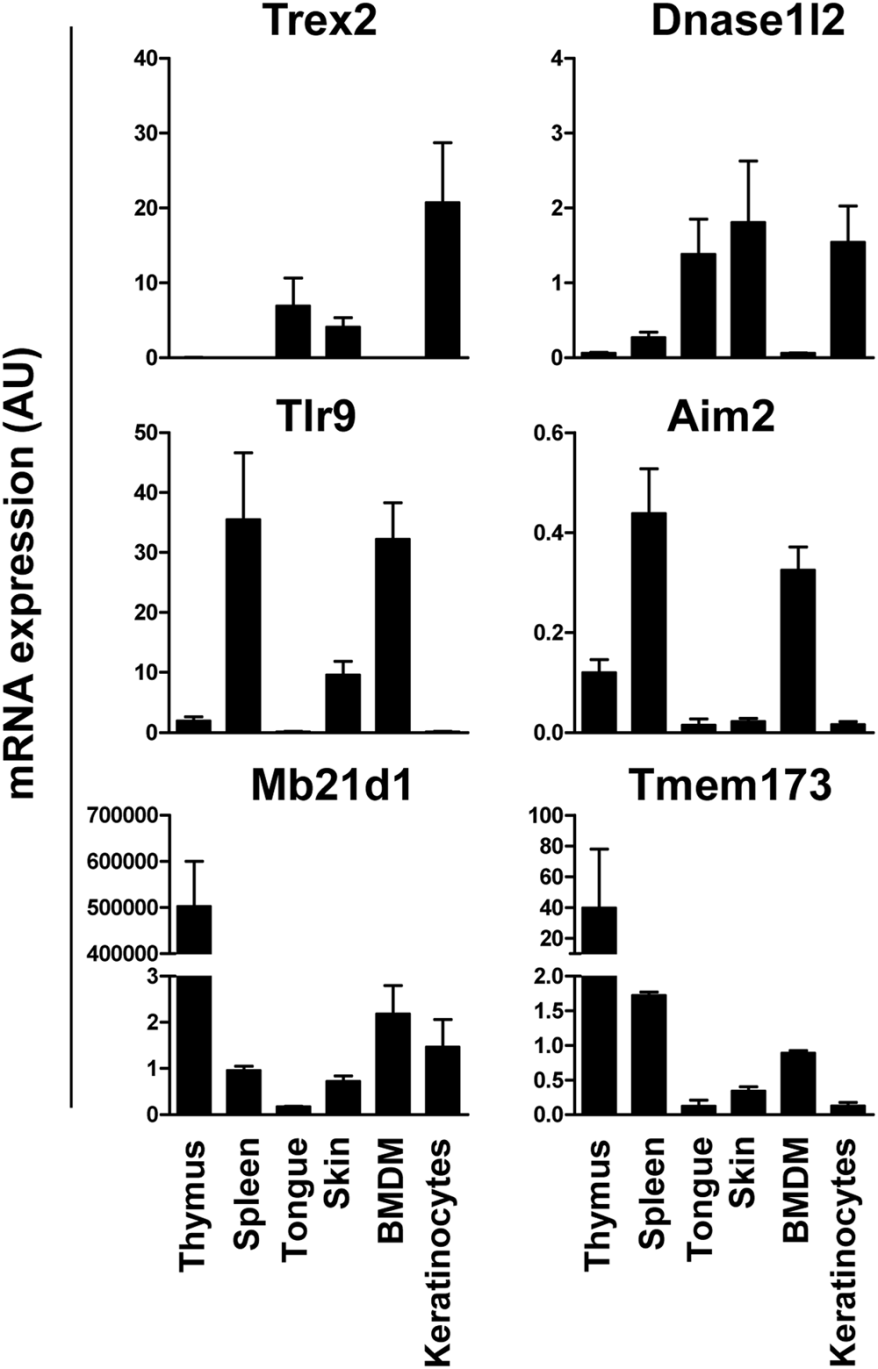
DNA sensing genes are poorly expressed in keratinocytes compared to immune cells, and in skin compared to tongue. Expression of the indicated key DNA sensing genes in mouse thymus, spleen, tongue, skin, bone marrow-derived macrophages (BMDM) and keratinocytes, as determined by RT-qPCR. Graphs show mean and standard error of the mean (SEM) of at least three biological samples. AU, arbitrary units.

**Figure 6:**
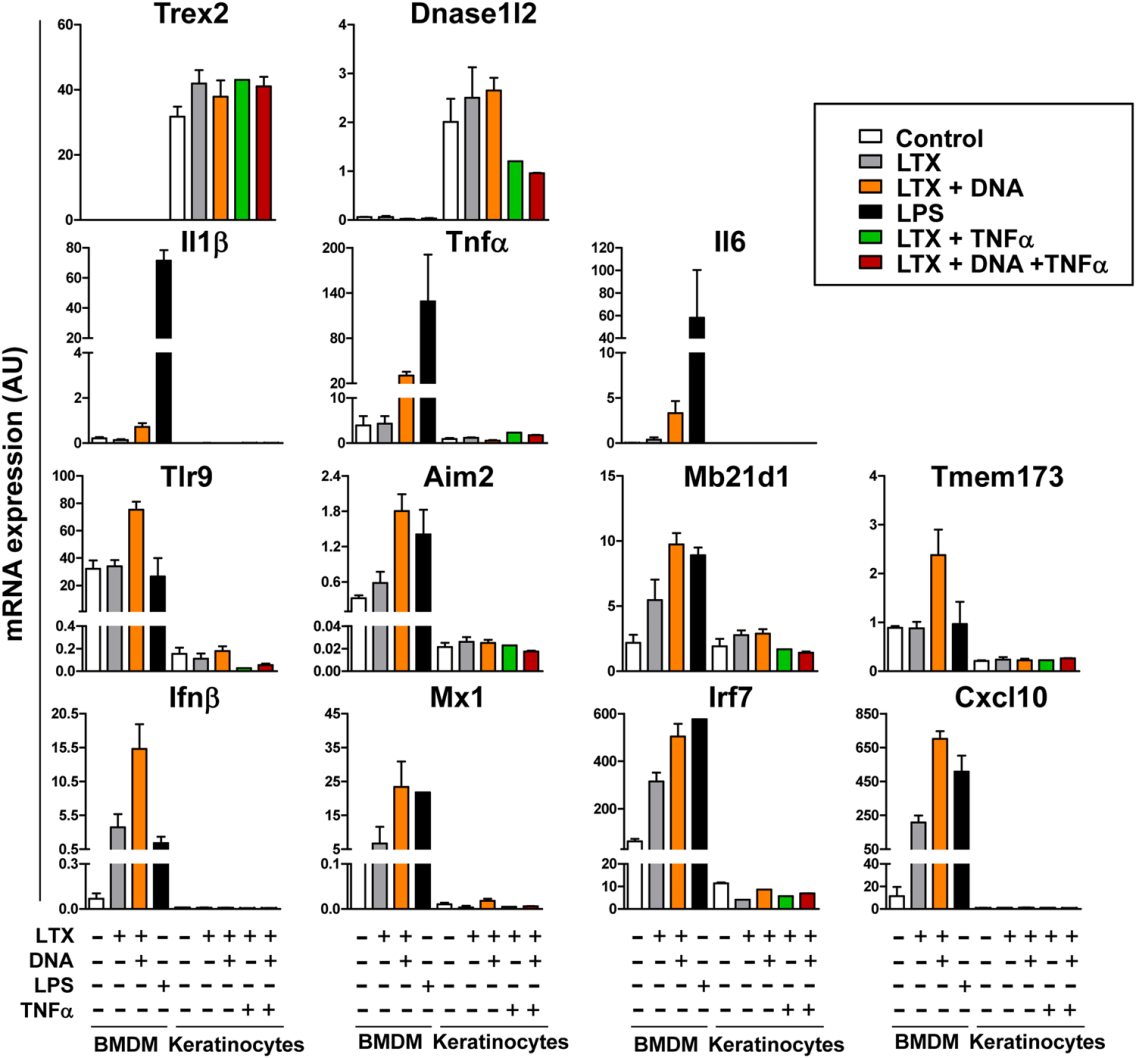
Intracellular DNA does not induce a transcriptional type I IFN response in mouse keratinocytes. Mouse bone marrow-derived macrophages (BMDM) and keratinocytes cells were untreated or treated with Lipofectamine® LTX (2 μl/ml), genomic DNA (2 μg/ml, premixed with Lipofectamine® LTX), LPS (10 ng/ml), TNFα (50 ng/ml) or a combination of these agents as indicated for 6 h prior to lysis. Total RNA was isolated, and mRNA levels of the indicated genes were quantified by RT-qPCR. Graphs show mean and SEM of at least three samples for each condition. The results from one of two independent experiments with similar results are shown. AU, arbitrary units.

Because the activation of the type I IFN response relies primarily on nucleic acid recognition by innate immune sensors, we tested whether the unresponsiveness to DNA accumulation in lingual *Trex2* ^−/−^ *Dnase1l2* ^−/−^ keratinocytes could be associated with differences in the expression levels of DNA-driven sensing and signalling genes relative to cell types with robust DNA response. First, we investigated the AIM2 and cGAS-STING pathways, which are the major cytosolic DNA-sensing pathways known to be activated by endogenous cytosolic DNA^36^. As shown in Fig. 5, the mRNA levels of *Aim2* and *Tlr9*, encoding key sensors for cytosolic and endosomal DNA respectively, and *Tmem173* (STING), were more than 10-fold to 100-fold lower in tongue, skin and keratinocytes, as compared to immune tissues and cells, such as thymus, spleen and bone marrow-derived macrophages (BMDM). The mRNA expression levels of *Mb21d1* (cGAS), a cytosolic DNA sensor upstream of STING, was hugely expressed in thymus compared to the rest of analysed samples revealing immune tissue-specific expression differences of key DNA signalling genes. STING was about 5-fold lower only in tongue but not in skin and keratinocytes, compared to spleen and BMDM. Notably, consistently with the lower inflammatory response observed in tongue compared to skin during wound healing^35^, the mRNA expression levels of these particular genes were lower in tongue than skin. In summary, at least Tlr9, Aim2 and STING, critical DNA sensing and signalling molecules, are expressed at relatively low levels in keratinocytes compared to BMDM, thereby likely intrinsically dampening DNA-driven responses in keratinocytes. Furthermore, consistent with the putatively reduced cell-intrinsic ability of keratinocytes to sense DNA and initiate a DNA-driven response, mouse keratinocytes were unresponsive to transfected DNA in comparison with BMDM (Fig. 6), similarly to what has been previously reported in human keratinocytes^37^. However, unlike human keratinocytes, TNFα stimulation did not render mouse keratinocyte responsive to cytosolic DNA (Fig. 6), thereby indicating another feature that distinguishes murine and human keratinocytes^38^. Taken together, our results suggest that mouse keratinocytes are intrinsically insensitive to cytosolic DNA, and this property may allow them to tolerate aberrant distribution of DNA in stratified epithelia such as that of the tongue.

## Discussion

The present study reveals that two keratinocyte-specific nucleases, the exonuclease Trex2 and the endonuclease DNase1L2, cooperate in the DNA degradation process occurring during keratinocyte terminal differentiation in the tongue epithelium. As schematically depicted in Supplementary Figure S6, our data identifies Trex2 as the critical exonuclease that degrades DNA from the 3′-OH free ends that have been previously generated by DNase1L2. *In vitro* TREX2 activity studies have demonstrated that TREX2 acts specifically on 3′-OH DNA ends^39,40^. Furthermore, ectopic co-expression of TREX2 with rare-cleaving endonucleases has shown that TREX2 processing of 3′-ends alter non-homologous end joining double strand DNA repair pathways^41–43^. In the absence of DNase1L2, even though DNA is cleaved by so far undetermined nucleases, nuclear DNA remains present in corneocytes where it is mainly retained inside the nucleus. By contrast, loss of Trex2 results in the accumulation of fragmented DNA in the cytosol of cornifying keratinocytes. Taking into account that simultaneous loss of Trex2 and DNase1L2 leads to synergistic deposition of DNA fragments in the cytosol and the nuclei, rather than a simple sum of single DNase1L2 and Trex2 deficiency signatures, our results clearly point at these two nucleases as members of the same DNA degrading pathway in the tongue. Probably, DNA degradation by Trex2 exonuclease activity is partially overlapped or compensated by the DNA breakdown activity of DNase1L2 endonuclease. In fact, a slight increase in DNase1L2 expression, even though it did not reach statistical significance (P = 0.128), may occur in Trex2-deficient skin (Fig. 4) that could partially compensate Trex2 loss. Thus, only when the two gene activities are missing, the cooperative interplay among them becomes visible in the tongue under homeostatic conditions.

Of note, although Trex2 and DNase1L2 activities seem to be dispensable for homeostatic epidermal skin cornification, it cannot be ruled out that they contribute, individually or coordinately, to DNA removal during epidermal cornification under certain stress conditions and pathological situations such as cancer and inflammatory and autoimmune diseases. In this context, it has been shown that Trex2 itself plays a crucial role in DNA degradation during keratinocyte death either via apoptosis or cornification processes in the hyperplastic epidermis of both psoriatic and ultraviolet B-treated skin^18–20^. In fact, TREX2 seems to be the only DNase that is strongly upregulated in both human and mouse psoriasis forms^20^. DNase1L2 and DNase2 have been found to cooperate in the enucleation of skin corneocytes^17^, whereas DNase2 alone is critical for nuclear DNA degradation of sebocytes^16^. Altogether, these findings support tissue keratinocyte-specific relevance of particular DNases in the DNA degradation processes associated with distinct modes of keratinocyte terminal differentiation.

The differential tissue keratinocyte-type dependent actions of Trex2 and DNase1L2 may be relevant in DNA degradation processes within various contexts. The mRNA levels of other nucleases do not seem to be regulated upon injury and did not differ between tongue mucosa and skin. In addition, caspase-14, a differentiation-associated gene reportedly involved in keratinocyte nuclear destruction^9,44,45^ is only upregulated during wound healing in the skin but not in the tongue. Hence, the transcriptional responses of lingual and epidermal epithelia following injury are different. Oral and skin epithelia also display different susceptibilities to tumorigenesis^46^, reflecting relevant differences among tongue and skin epithelia and keratinocytes at either the levels of cell turnover, differentiation, morphology and/or metabolism. Future studies should determine whether Trex2 and DNase1L2 may play distinct roles in the tongue and skin keratinocytes under stress and pathological conditions, and their putative interplay with other nucleases and signalling pathways.

The distribution of Trex2 and DNase1L2 proteins, as determined by immunofluorescence, in epithelial layers parallel the spatial and temporal pattern of the loss of the nucleus in keratinocytes, supporting a crucial role of these nucleases in keratinocyte nuclear DNA degradation. At the mRNA level, the comparative analysis of basal, early spinous, late spinous and granular layer transcriptomes of murine epidermis^32^ uncovered that among known nucleases involved in intrinsic and extrinsic DNA degradation, *Trex2* and *Dnase1l2* were specifically and highly upregulated in the granular layer (Supplementary Figure S1), in agreement with their immunostaining patterns in skin epidermal layers (Fig. 1), while no major changes were observed in the mRNA abundance of *Dffb/CAD*, *Dnase1*, *Dnase1l1*, *Dnase1l3*, *Dnase2a*, and *Trex1*. Noteworthy, lingual cornification-associated DNA degradation can neither be compensated by ubiquitous Trex1, closely related to Trex2^31^ and critically implicated in cytoplasmic DNA degradation^47,48^, nor Dffb/CAD, the main cell-autonomous DNase activated by caspase-3 that cleaves DNA in apoptotic cells^49^. Nevertheless, overlap and cooperation may take place, depending on tissue and pathways engaged to maintain homeostasis. For example, it has been proposed that DFFB/CAD is activated by caspase-14 and thus contributes to DNA degradation in differentiating human keratinocytes^9^. Caspase-14 is specifically upregulated (Supplementary Figure S1) and activated^34,44,50,51^ during keratinocyte differentiation and required for complete epidermis cornification, especially when its homeostasis is challenged^7,45^.

The absence of detectable DNA-driven immune responses against the huge accumulation of cytosolic self-DNA in the lingual epithelium of *Trex2* ^−/−^ *Dnase1l2* ^−/−^ mice, along with the mouse keratinocyte unresponsiveness to transfected DNA, as was also recently observed in human keratinocytes^37^, indicate that epithelia and keratinocytes, in particular, are highly tolerant to DNA. Accordingly, deletion of *Trex1* in dendritic cells but not in keratinocytes leads to DNA-driven autoimmune phenotype in mice^29^. One explanation would be related to the level of expression of key DNA sensing and signalling genes. In line with this hypothesis, we showed that the mRNA expression amounts of *Tmem173* (STING), which mediates type I IFN response to intracellular DNA^52,53^, were much lower in murine keratinocytes and keratinocyte-built epithelial tissues in tongue and skin, compared to macrophages and immune tissues. Consistently, *Ifnβ* and some key DNA-driven genes in the distinct keratinocyte layers were shown to be expressed at very low levels in mouse epidermal keratinocyte layers, and, with the notable exception of *Irf7*, were not transcriptionally induced (*Aim2*, *Mb21d1*, *Mx1*) or were downregulated (*Cxcl10*, *Irf9*, *Tmem173*) during keratinocyte differentiation in upper layers (Supplementary Figure S1). It has been suggested that low constitutive expression of type I IFN response genes in epithelial surfaces, which are naturally exposed to exogenous DNA, might be a strategy to avoid premature and excessive immune response^54^. Complementarily to the putatively low DNA-responsiveness of the keratinocytes, the lack of DNase1L2 and Trex2 may not only compromise degradation but also sensing of DNA in *Trex2* ^−/−^ *Dnase1l2* ^−/−^ tongue. In fact, Trex2 deficiency leads to attenuated skin inflammatory response to both imiquimod-induced psoriasis^20^ and ultraviolet-B radiation^19^. Furthermore, increasing evidence indicates supportive roles of distinct nucleases in nucleic acid immunity. The sensing of DNA by TLR9 requires digestion by DNase-II^55^, and short rather than large DNA fragments are efficient inflammasome activators^56^. Alternatively, immunostimulatory properties of DNA could be physically dampened as keratinocytes are committed to cornification. In particular, the formation of a cornified envelope may structurally prevent the release of DNA from terminally differentiated keratinocytes and, thus, its engulfment and sensing by dendritic cells and other immune cells that trigger potent immune responses to DNA. In contrast to keratinocytes, skin immune cells can be efficiently activated by endogenous nucleic acids, such as aberrant replicative DNA structures or damaged DNA, that may be released by highly proliferative or stressed keratinocytes and subsequently drive the onset and pathogenesis of skin inflammatory diseases, such as cutaneous lupus erythematosus^57,58^.

Prior to cornification-associated degradation of DNA, the organization of chromatin changes during terminal keratinocyte differentiation^59,60^. Thus it is conceivable that Trex2 and DNase1L2 mediate limited degradation of DNA during chromatin reorganization in differentiated keratinocytes when they are still alive. Potentially, such activities may influence gene expression during the final stages of keratinocyte differentiation. Interestingly, by means of mRNA sequencing, genes related to skin differentiation and chromatin biology, among others, are differentially expressed in Trex2 deficient imiquimod-treated skin as compared to treated wild-type skin^20^. Possible roles, besides DNA degradation in dying cells, of DNase1L2 and Trex2 deserve further studies.

In summary, we have shown that the Trex2 exonuclease and the DNase1L2 endonuclease, which are expressed in parallel to keratinocyte enucleation, cooperate and contribute to DNA degradation during keratinocyte terminal differentiation. Our demonstration of accumulating DNA fragments in the lingual epithelium of Trex2/DNase1L2-double deficient mice suggests not only a mechanism of DNA breakdown but, due to the absence of epithelial inflammation, also supports the concept of physiological tolerance of keratinocytes to intermediates of cornification-associated processing of endogenous DNA.

## Methods

### Mice

*Trex2* ^−/−^ mice on C57BL/6 N background^18^ and *Dnase1l2* ^−/−^ mice on 129SvEv^14^ have been described previously. The *Trex2* ^−/−^ *Dnase1l2* ^−/−^ mice were generated by crossing the *Trex2* ^−/−^ and *Dnase1l2* ^−/−^ mice. *Dnase1l2* ^−/−^ mice and *Trex2* ^−/−^ *Dnase1l2* ^−/−^ mice were backcrossed eight times to C57BL/6 N background. The *Trex2* ^−/−^, *Dnase1l2* ^−/−^, *Trex2* ^−/−^ *Dnase1l2* ^−/−^ and wt C57BL/6 mice were housed and bred under specific pathogen-free environment in the animal facility of Parc Científic of Barcelona, University of Barcelona, according the institutional Ethics Committee on Animal Care. All animal experiments and methods were conducted under the authorizations of the Catalan Government (DAAM 7894 and DAAM 7895), and were performed in accordance with the relevant guidelines and regulations. Analyses were undertaken in 2 to 6-day old newborn mice, and 6- to 9-week old adult male and female mice, as indicated.

### Histological and immunostaining analysis

Tissue samples from mice were fixed with 4% paraformaldehyde, embedded in paraffin and then cut in 5 μm sections. Sample processing for hematoxylin and eosin (H&E), staining and immunofluorescence assays was performed according to standard procedures^33^. For Trex2 immunofluorescence labelling, tissue sections were deparaffinised and rehydrated, and antigen retrieval was performed in Tris-EDTA buffer (pH 9.0). After blocking, sections were incubated with affinity-purified rabbit anti-mouse Trex2 (1/50^18^). Goat anti-rabbit IgG coupled to Alexa Fluor 555 (1/500; Life Technologies) was used as secondary antibody. Nuclei were counterstained with DAPI (Sigma), and sections were mounted in ProLong Gold Anti-Fade reagent (Life Technologies). For DNase1L2 immunofluorescence labelling, tissue sections were deparaffinised and rehydrated, and antigen retrieval was performed in citrate buffer pH6 (Dako). Sections were incubated with affinity-purified rabbit anti-DNase1L2 (1/100^14^). Goat anti-rabbit IgG coupled to Alexa Fluor 546 (1/500; Molecular Probes) was used as secondary antibody. Nuclei were counterstained with Hoechst 33258 (Sigma), and sections were mounted with Fluoprep (Biomerieux). Conventional images were captured using a Nikon Eclipse E-800 fluorescence microscope and an Olympus AX 70 microscope (Hamburg, Germany).

### DNA labelling

The TUNEL (Terminal deoxynucleotidyl transferease dUTP nick end labeling) assay was performed to label 3′-OH ends of DNA *in situ*, according to the kit manufacturer’s instructions (*In Situ* Cell Death Detection Kit, TMR Red; Roche). Nuclear DNA was counterstained with Hoechst 33258 (Sigma). Slices were mounted onto glass slides using Fluoprep (bioMérieux, Marcy l′Etoile, France). Images were captured using an Olympus AX 70 microscope (Hamburg, Germany) equipped with a Spot RT3 slider camera (SPOT Imaging Solutions, Sterling Heights, MI).

### Cells and treatments

Primary mouse keratinocyte cell cultures were prepared from 2 days-old newborn mouse skin and cultured in CNT-02 medium (CELLnTEC) for up to seven days, as previously described^18^, in either 24 or 12 wells plates. The medium was changed every second day. Cells were used for experiments at day 7 post plating. Bone marrow derived macrophages (BMDMs) from mice were cultured in DMEM supplemented with 20% fetal bovine serum and 30% supernatant of L929 cell conditioned media as a source of M-CSF, as previously described^61^. Macrophages were obtained as a homogeneous population of adherent cells after 7 days of culture. Then, cells were scrapped, counted and seeded for experiments in either 24 or 12-wells plates. Cells were left untreated, transfected with genomic DNA (2 μg/ml) using Lipofectamine® LTX (2 μl/ml; Invitrogen) or stimulated with 10 ng/ml of LPS (Sigma-Aldrich), 50 ng/ml TNFα (PreproTech Inc) or a combination of these agents, as indicated. Genomic DNA from mouse skin, treated with Proteinase K and RNase A, was purified using a silica-based column DNA isolation kit for tissues (Biotools B&M Labs), according to manufacturer’s indications, and eluted with water. For treatments, DNA was pre-incubated with Lipofectamine® LTX according to the manufacturer’s instructions. All cell treatments were left for 6 hours and then lysis was performed to extract total RNA.

### RNA quantitative analysis

Total RNA from tissues and cells was isolated using the NucleoSpin RNA extraction kit (Macherey-Nagel), quantified and analysed using a NanoDrop ND-1000 Spectrophotomer, and cDNA synthesis was done using the GeneAmp RNA PCR kit (Applied Biosystems). Quantitative PCR (qPCR) amplification reactions were performed in the 7500 Fast Real-Time PCR System (Applied Biosystems). TaqMan Gene Expression Master Mix and predesigned probes (Applied Biosystems) were used to quantify mRNA expression of the following genes: *Trex2* (Mm04210320_m1), *Dnase1l2* (Mm00481868_g1), *Ifnβ* (Mm00439552_s1), *Tnf*α (Mm00443258_m1), *Cxcl10* (Mm00445235_m1), *Mx1* (Mm00487796_m1), Aim2 (Mm01295719_m1), IL6 (Mm00446190_m1), IL1β (Mm00434228_m1), and *Sdha* (Mm01352366_m1). Power SYBR Green PCR Master Mix (Applied Biosystems) and the following gene primers ere used to quantify mRNA expression of the following genes: *Irf7* Forward (F) 5′GAGCAAGACCGTGTTTACG and Reverse (R) 5′CATGATGGTCACATCCAGG; *Irf9* (F) 5′CAGTGTTCCTGGAGCATCAA and (R) 5′ACGCCTCTGTCAAGCTGATT; *Tlr9* (F) 5′GAATCCTCCATCTCCCAACATG and (R) GAGGCTTCAGCTCACAGGG; *Mb21d1* (F) 5′GTCGGAGTTCAAAGGTGTGGA and (R) 5′GACTCAGCGGATTTCCTCGTG; and *Tmem173* (F) 5′CTACATTGGGTACTTGCGGTT and (R) 5′GCACCACTGAGCATGTTGTTATG. The results are presented relative to those for the housekeeping gene S*dha* using the ΔCq method.

Supplementary Figures S1 and S2 show the processed transcriptomic data available from GSE23006 and GSE75931 respectively plotted in R.

### Analysis of antinuclear antibodies

Human epithelial (HEp-2) cell substrates slides (INOVA Diagnostics) and goat anti-mouse IgG coupled to Alexa Fluor 488 (1/500; Life Technologies) was used to assess the presence of antinuclear antibodies (ANAs) in mouse sera, following the manufacturer’s instructions. Serum was obtained from blood that was extracted by maxillary punction from 9-weeks old mice, allowed to coagulate at room temperature, and then centrifuged. Supernatant was collected and stored at −80 °C for further ANA analysis. Immunofluorescence patterns were examined using a Nikon Eclipse E-800 fluorescence microscope at a magnification of 400X. An Immunodot Kit (BlueDot, D-tek) was used for the detection in mouse sera of IgG autoantibodies against nucleosomes and histones.

### Statistical analysis

The Mann-Whitney test was applied for comparisons between genotypes. Test was two-tailed. The *P* values of ≤0.05 were considered to be statistically significant. Analysis was performed using GraphPad Prism software.

### Data availability statement

The datasets generated and analysed during the current study are available from the corresponding authors on reasonable request.

## Electronic supplementary material

Supplementary Information
