# Supplementary material for: Double deficiency of Trex2 and DNase1L2 nucleases leads to accumulation of DNA in lingual cornifying keratinocytes without activating inflammatory responses
Source: Sci Rep. 2017 Sep 19;7:11902. doi: 10.1038/s41598-017-12308-4 (PMC5605544; doi:10.1038/s41598-017-12308-4)
Supplement: Supplementary file 1 — Supplementary Information [file 41598_2017_12308_MOESM1_ESM.pdf]

SUPPLEMENTARY INFORMATION

Double deficiency of Trex2 and Dnase1L2 nucleases leads to accumulation of DNA in lingual cornifying keratinocytes without activating inflammatory responses

Joan Manils[1#&], Heinz Fischer[2,#,§], Joan Climent[3], Eduard Casas[4], Celia García-Martínez[1], Jordi Bas[3], Supawadee Sukseree[2], Tanya Vavouri[4,5], Francisco Ciruela[1], Josep Maria de Anta[1], Erwin Tschachler[2], Leopold Eckhart[2*] and Concepció Soler[1*]

[1]Departament de Patologia i Terapèutica Experimental, Facultat de Medicina i Ciències de la Salut, IDIBELL, Universitat de Barcelona, L'Hospitalet de Llobregat, Barcelona, Spain

[2]Research Division of Biology and Pathobiology of the Skin, Department of Dermatology, Medical University of Vienna, Vienna, Austria

[3]Departament d'Immunologia, Hospital Universitari de Bellvitge, L'Hospitalet de Llobregat, Barcelona, Spain.

[4]Program of Predictive and Personalized Medicine of Cancer (PMPPC) - Institute Germans Trias i Pujol (IGTP), Badalona, Barcelona, Spain

[5]Josep Carreras Leukaemia Research Institute (IJC), ICO-Hospital Germans Trias i Pujol, Badalona, Barcelona, Spain

[#] contributed equally

[&]The Francis Crick Institute-Mill Hill Laboratory, London, NW7 1AA, United Kingdom

[§]Current address: Unit of Pathology of Laboratory Animals, University of Veterinary Medicine, Vienna, Austria

* co-corresponding

Supplementary Figure S1. Expression of DNases, caspases and DNA-driven genes in the basal, early spinous, late spinous and granular layers from mouse epidermis. Expression of the indicated DNase, caspase and DNA-driven genes using the public transcriptome dataset GSE75931 (Asare et al., 2017). mRNA expression values are normalized fragments per kilobase of transcript per million mapped reads.

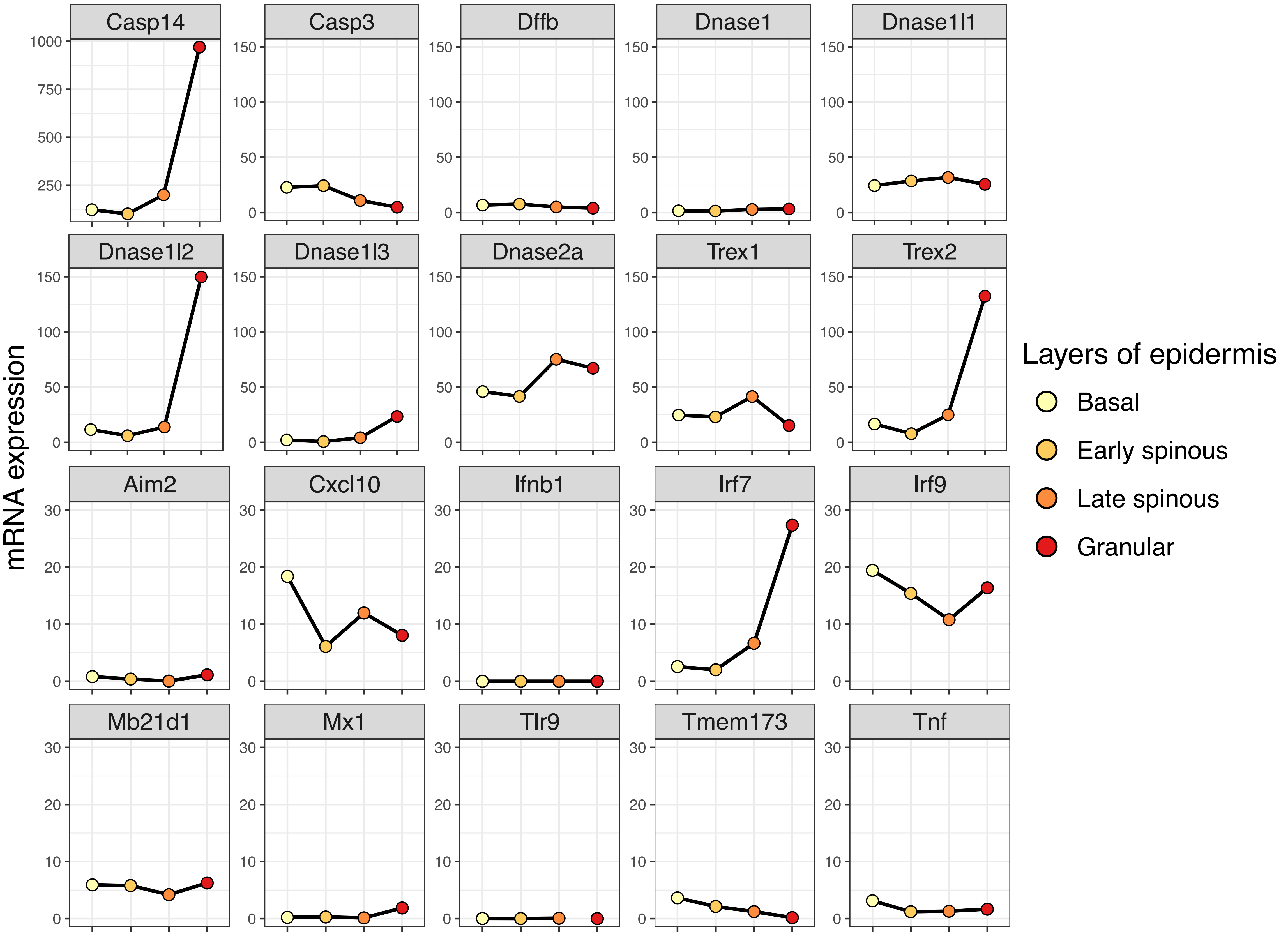

Supplementary Figure S2. Differential expression of DNases and caspases in mouse tongue and skin during wound healing. Expression of the indicated DNase and Caspase genes in tongue and skin, at the indicated times after wounding using the public available microarray dataset GSE23006 (Chen et al., 2010). mRNA expression values are normalised microarray expression data. Graphs show the gene expression values by the mean and SEM of three individual samples on each condition.

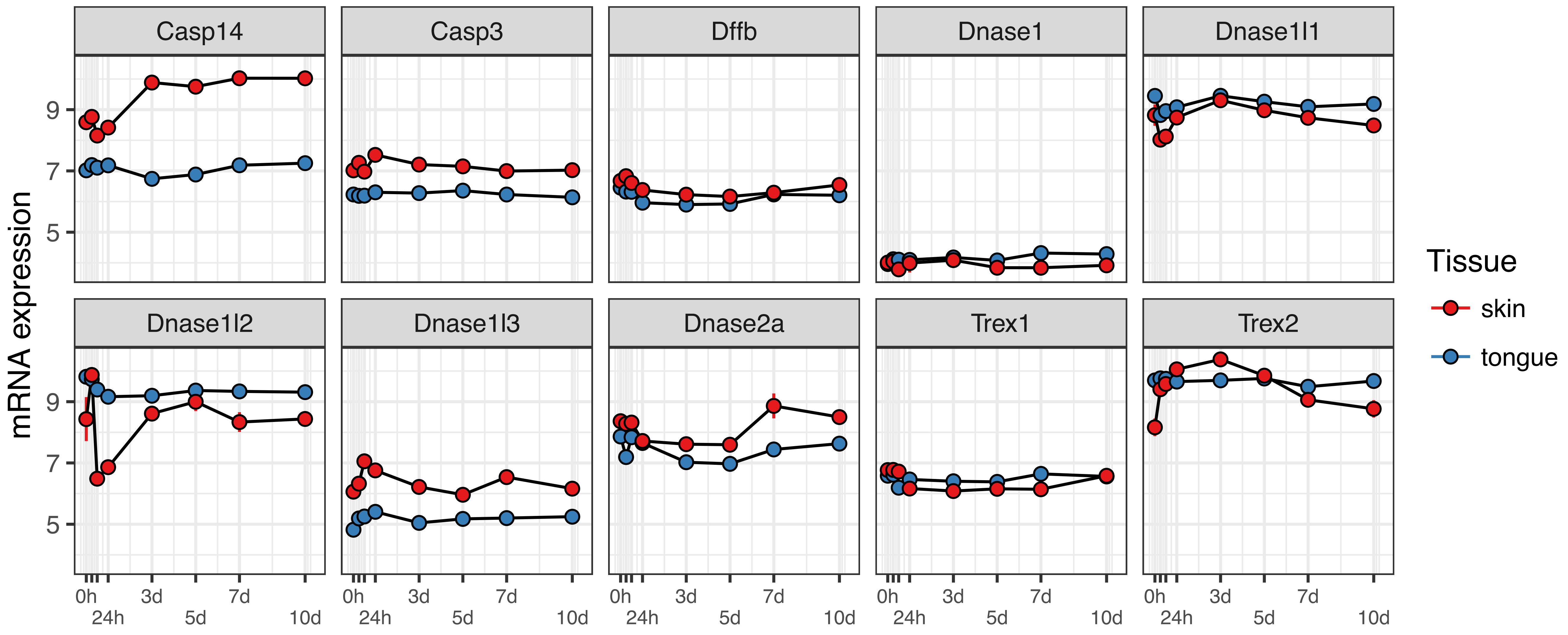

Supplementary Figure S3. Double *Trex2* and *Dnase1l2* deficiency does not trigger parakeratosis in the ear and snout skin.

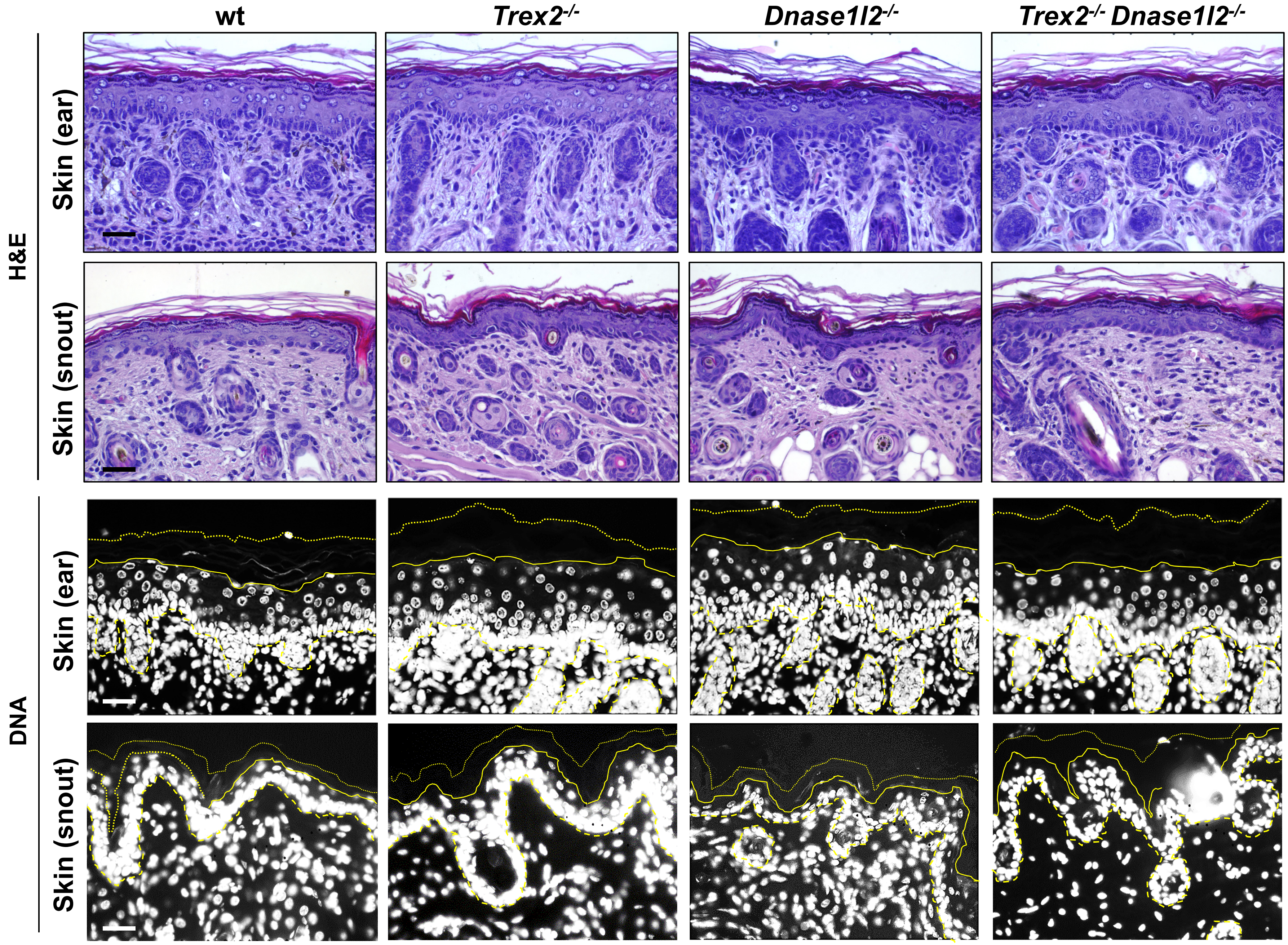

Supplementary Figure S3. Double *Trex2* and *Dnase1l2* deficiency does not trigger parakeratosis in the ear and snout skin. H&E (lanes 1 and 2) staining and Hoechst DNA labelling (lanes 3 and 4) in ear and snout skin sections from wt, *Trex2*$^{-/-}$, *Dnase1l2*$^{-/-}$ and *Trex2*$^{-/-}$*Dnase1l2*$^{-/-}$ mice. Samples were from adult 7-9 weeks-old mice Representative images of at least four mice from each genotype. Scale bars = 25 μm. Dashed lines, epidermal-dermal border; continuous lines, bottom border of the stratum corneum; dotted lines, upper border of the stratum corneum.

Supplementary Figure S4. Double Trex2 and Dnase1l2 deficiency does not lead to changes in the expression of keratinocyte differentiation genes. Expression of the indicated genes in the tongue from wt, *Trex2*$^{-/-}$, *Dnase1l2*$^{-/-}$ and *Trex2*$^{-/-}$*Dnase1l2*$^{-/-}$ mice, as determined by RT-qPCR. Each dot indicates a sample from an individual mouse, and horizontal lines represent the mean value.

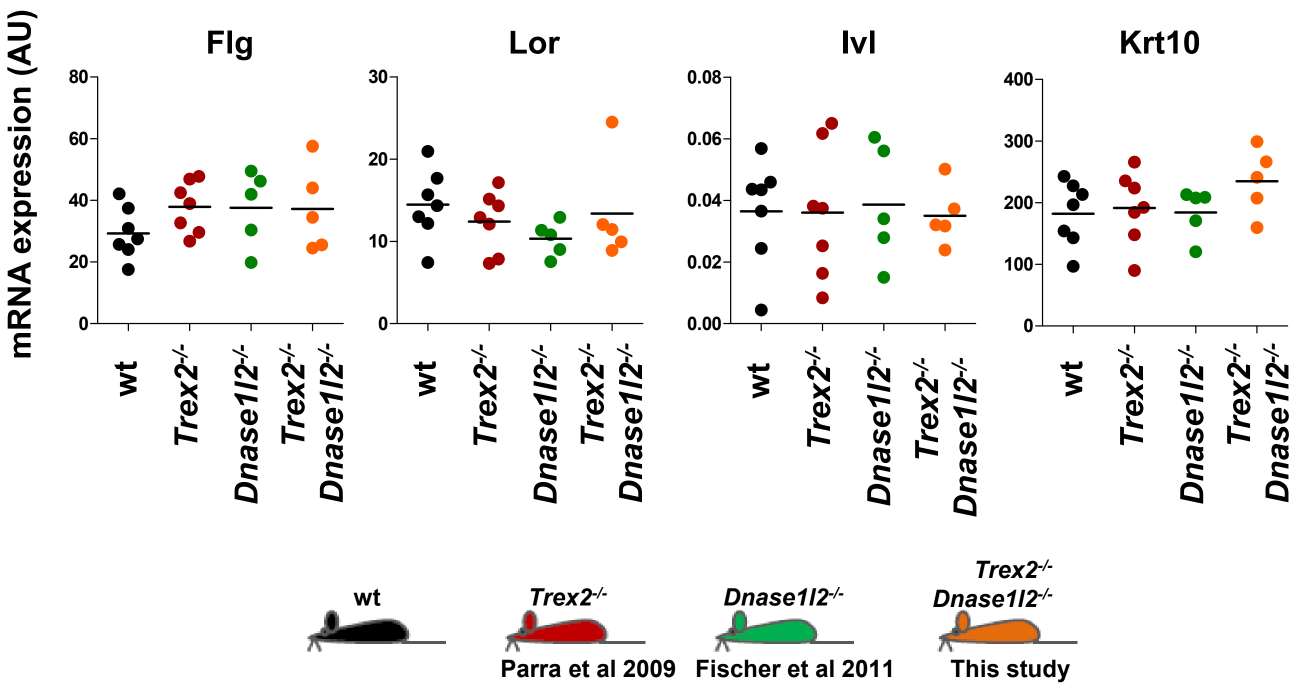

Supplementary Figure S5. Absence of antibodies against nuclear antigens in the *Trex2*$^{-/-}$ *Dnase1l2*$^{-/-}$ mice. Representative pictures obtained from the immunofluorescence analysis assay of antinuclear antibodies (ANAs) in serum from wt, *Trex2*$^{-/-}$, *Dnase1l2*$^{-/-}$ and *Trex2*$^{-/-}$*Dnase1l2*$^{-/-}$ mice using the HEp-2 human epithelial cell substrate slides, as indicated in Methods. Serums were obtained from adult 9 weeks-old mice. Number (n) of positive and negative serums for ANAs from each genotype is indicated. NUMA, nuclear mitotic apparatus. Magnification 400x.

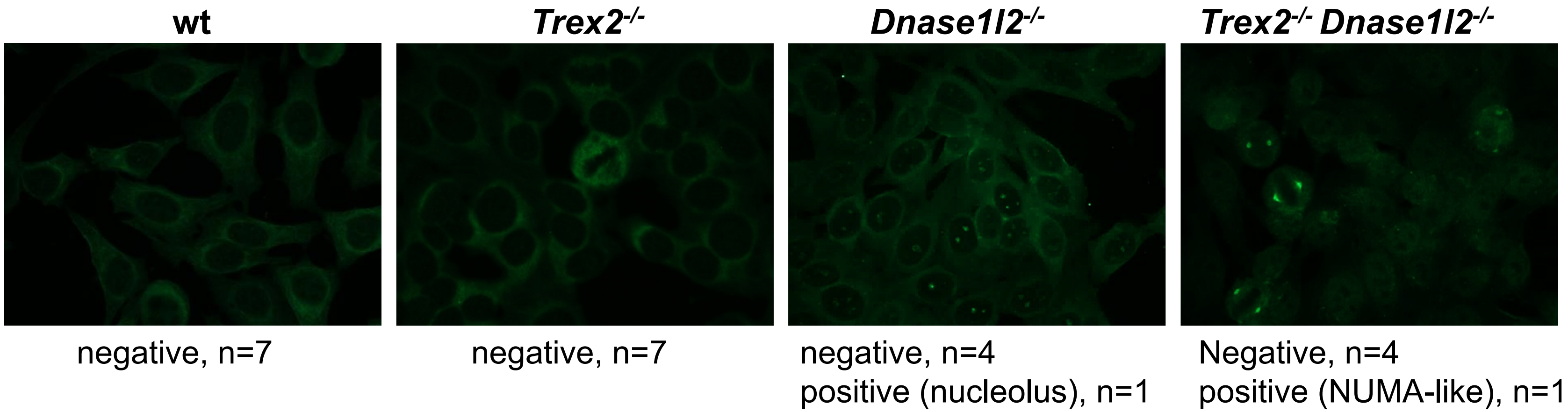

Supplementary Figure S6. Schematic diagram of DNA degradation in cornifying keratinocytes of the tongue. The interplay of DNase1L2 and Trex2 in DNA degradation during cornification of lingual keratinocytes is schematically summarized. The results of this study suggest that DNA is first fragmented by the endonucleolytic activity of DNase1L2 and subsequently degraded by the exonuclease Trex2. At least one more DNA fragmentation process (not depicted here) is active to generate DNA fragments with 3´-OH ends in *Trex2*$^{-/-}$ Dnase1l2$^{-/-}$ mice (Figure 3).

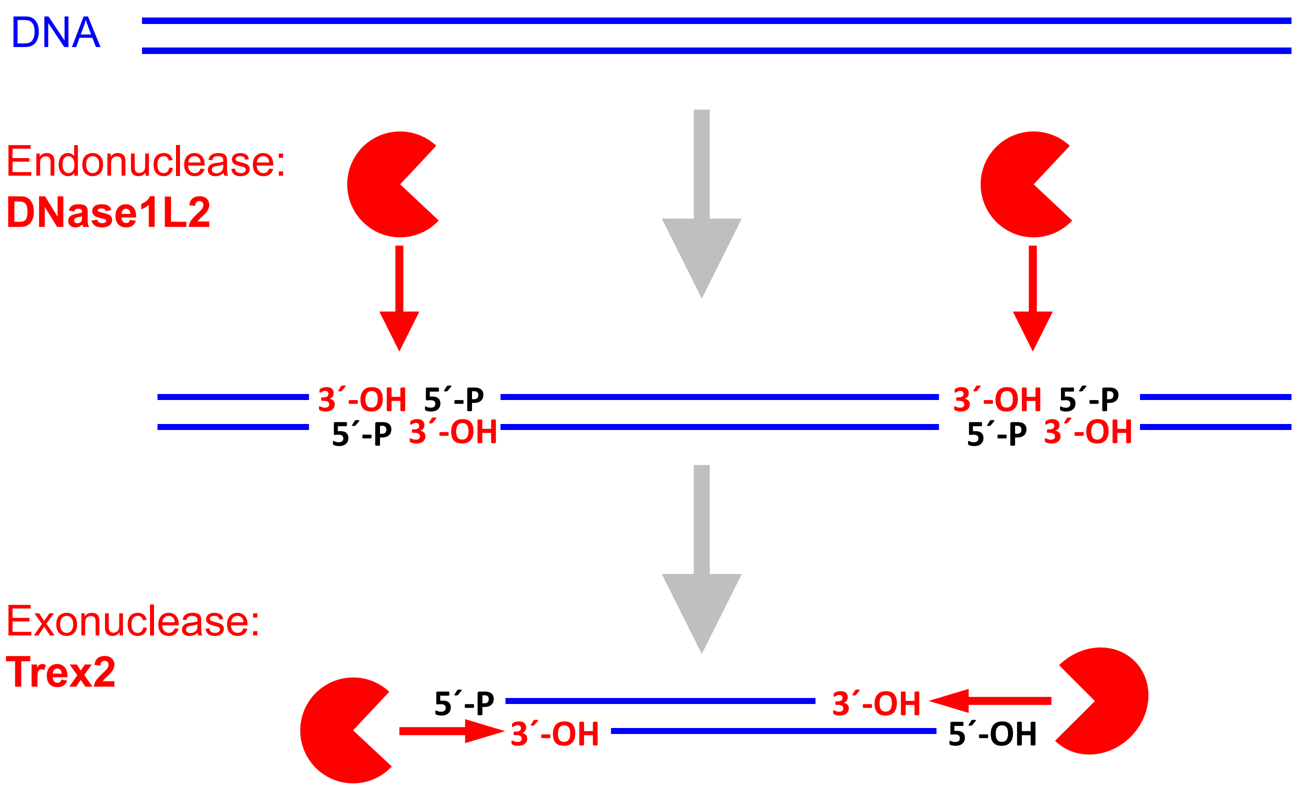

REFERENCES

Asare, A., Levorse, J. & Fuchs, E. Coupling organelle inheritance with mitosis to balance growth and differentiation. *Science* **355** (2017).

Chen, L. *et al.* Positional differences in the wound transcriptome of skin and oral mucosa. *BMC Genomics* **11**, 471 (2010).
